# Risk factors for cough after pulmonary resection

**DOI:** 10.1186/s12957-023-03235-y

**Published:** 2023-11-03

**Authors:** Yongming Wu, Wenpeng Song, Dongmei Zhu, Yan Wang, Guowei Che

**Affiliations:** 1 Department of Thoracic Surgery, West China Hospital, Sichuan University, Chengdu, Sichuan China; 2 Lung Cancer Center, West China Hospital, Sichuan University, Chengdu, Sichuan China; 3 Bengbu Medical College, Bengbu, Anhui China; 4 The People’s Hospital of Bozhou, Bozhou, Anhui China

**Keywords:** Pulmonary resection, Cough, Risk factor, Surgery

## Abstract

**Background:**

To investigate the risk factors for cough after pulmonary resection.

**Methods:**

The PubMed, Embase, Web of Science, ClinicalTrials.gov, and China National Knowledge Network databases were searched from inception to November 2022. The *Q* tests and *I*^*2*^ statistic were used to evaluate the heterogeneity. Odds ratios (OR) were combined using the inverse variance method. All statistical analyses were performed by RevMan 5.4.1.

**Results:**

Nineteen studies with 4755 patients were included, the incidence of postoperative cough was 21.1%-55.8%. The results showed that young age [OR = 0.66, 95% CI (0.46, 0.96), *p* = 0.03], female sex [OR = 1.69, 95% CI (1.07, 2.66), *p* = 0.02], preoperative cough [OR = 5.96, 95% CI (2.58, 13.73), *p* < 0.01], right lobe operation [OR = 2.14, 95% CI (1.44, 3.19), *p* < 0.01], lobectomy [OR = 3.70, 95% CI (1.73, 7.90), *p* < 0.01], subcarinal lymph node dissection [OR = 3.45, 95% CI (1.86, 6.39), *p* < 0.01], mediastinal lymph node removal [OR = 3.49, 95% CI (2.07, 5.89), *p* < 0.01], closure of bronchial stump with stapler [OR = 5.19, 95% CI (1.79, 15.07), *p* < 0.01], peritracheal lymph node resection [OR = 3.05, 95%CI (1.40,6.64), *p* < 0.01], postoperative acid reflux [OR = 11.07, 95%CI (4.38,28.02),* p* < 0.01] were independent risk factors for cough after pulmonary resection.

**Conclusions:**

Young age, female sex, preoperative cough, right lobe operation, lobectomy, subcarinal lymph node dissection, mediastinal lymph node removal, closure of bronchial stump with stapler, peritracheal lymph node resection, and postoperative acid reflux are independent risk factors for cough after pulmonary resection.

## Background

**Fig. 1:**
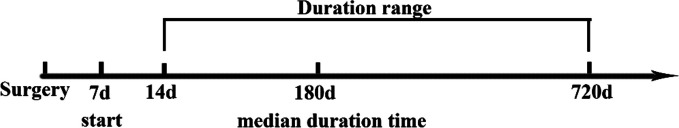
The natural history of postoperative cough. Cough after pulmonary resection typically arose around the first postoperative week. The median duration of persistent cough after pulmonary resection was approximately 180 days, ranging from 14 to 720 days

Several studies have shown that persistent cough was one of the most common complications after pulmonary resection, with an incidence of 25%-50% [1–3]. In most studies, cough after pulmonary resection (CAP) was defined as follows: (1) no obvious cough history before surgery; (2) postoperative cough occurred within 30 days after surgery and lasted no less than two weeks; (3) exclude tumor recurrence; (4) cough caused by postoperative infection and other medical factors was excluded [3]. Our previous studies indicated that pain and cough were the main symptoms after pulmonary resection. The occurrence of cough was delayed, with a low incidence at the time of discharge, peaked 30 days after discharge, and turned to mild or disappeared more than 90 days after discharge [1]. Mu et al. found that CAP mostly started on the 6th day after surgery, with a median duration of 180 days (range 14–720 days) (Fig. 1) [4]. Persistent cough after surgery can increase incision pain and interfere with sleep and conversation, thereby reducing patients' quality of life [5, 6]. Due to the lack of guidance from professional doctors after discharge, cough after discharge may hinder the recovery of patients, hinder the return of patients to daily life. In recent years, the management of postoperative cough has gradually gained attention, due to the spread of the concept of enhanced recovery after surgery (ERAS).

Some studies have confirmed that CAP may be associated with vagus nerve injury, lymph node dissection, duration of anesthesia, and gastroesophageal reflux. However, due to the different risk factors included in various studies and the differences in results, the risk factors for CAP are still controversial [2, 4, 7, 8]. Therefore, to further explore the risk factors for CAP, we conducted this meta-analysis. To our knowledge, this is the first meta-analysis to explore the risk factors for CAP.

## Materials and methods

This meta-analysis was presented according to the Preferred Reporting Items for Systematic Reviews and Meta-Analyses statement. Our study was registered in the International Prospective Registry of Systematic Reviews (CRD42022360462).

### Literature research

Relevant literature in the PubMed, Embase, Web of Science, ClinicalTrials.gov, and CNKI databases were searched, the retrieval time was from the establishment of the database to November 2022. The key terms used were: thoracic surgery, video-assisted thoracic surgery, pulmonary resection, pneumonectomy, wedge resection, segmentectomy, lobectomy, sublobar resection, sublobectomy, sleeve lobectomy, cough, etc. Additionally, references of all included studies and relevant review articles were searched for available articles.

### Inclusion and exclusion criteria

#### Inclusion criteria

(1) participants: patients who underwent minimally invasive or open lung resection; (2) cohort study or case–control study; (3) Studies that looked at postoperative cough as the primary outcome; (4) OR and corresponding 95% confidence intervals (CI) were provided; (5) risk factors reported in two or more studies.

#### Exclusion criteria

(1) incomplete data, duplication or complete data cannot be obtained; (2) conference abstracts, case reports, reviews; (3) repeatedly published literature.

### Data extraction and quality assessment

Two authors (WYM and SWP) independently screened the literature, and any differences in the research process were discussed through the team. The extracted information included: the first author, publication year, country, study type, sample size, incidence of postoperative cough, TNM stage, operation method, the definition of CAP, OR and responding 95% CI. Additionally, during the data extraction process, we assessed the definition of CAP in each article. If an article did not provide a definition for CAP, it was required to clarify how CAP was assessed within the text, or else it would be excluded from consideration. Literature quality was evaluated by the Newcastle–Ottawa Quality Scale (NOS), and studies with a NOS score of 6 or higher were regarded as high-quality studies.

### Statistical analysis

All statistical analyses were performed using RevMan (version 5.4.1, the official software of the Cochrane Collaboration Group). The OR values and 95% CI of the multivariate analysis of CAP were extracted, and the OR values were combined using the inverse variance method. The *Q* tests and *I*^*2*^ statistic were used to evaluate the heterogeneity among the included references. If *I*^*2*^ ≥ 50% or (and) *p* ≤ 0.10, the random-effects model was used; otherwise, the fixed-effects model was used. A funnel plot was used to assess publication bias for risk factors with ≥ 10 articles included.

## Results

**Fig. 2:**
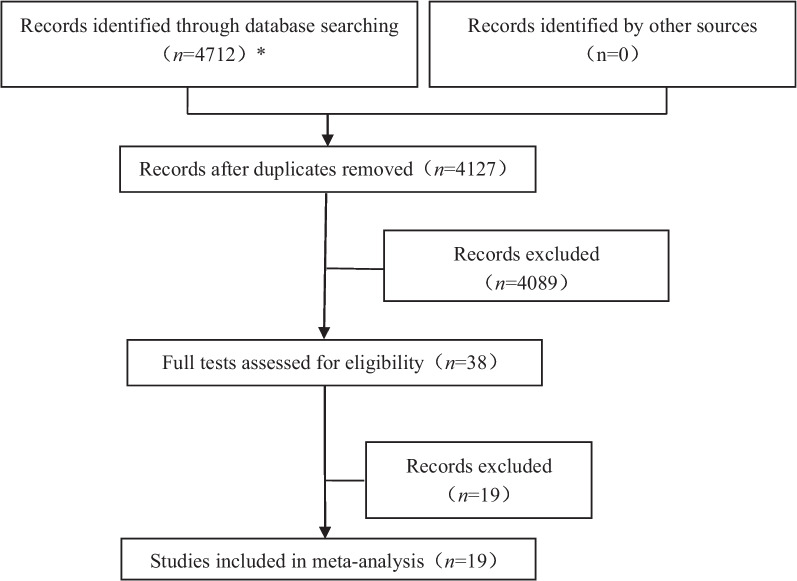
Flow diagram of the meta-analysis. *EMbase (*n* = 1751), PubMed (*n* = 455), Web of Science (*n* = 2086), ClinicalTrials.gov (*n* = 100), CNKI (*n* = 320)

Based on the research strategy, 4712 relevant studies were retrieved, and 19 studies were included in the meta-analysis after gradual screening. The selection process is shown in Fig. 2. Finally, nineteen case–control studies involving 4755 patients were included [2–4, 7–22], among them, 1535 patients suffered postoperative cough, the incidence of postoperative cough was 21.1%-55.8%, and a total of 18 independent risk factors were included. The detailed characteristics of the included studies are presented in Table 1. The definitions of CAP for each study were shown in Table 1. All of the included studies had an NOS score of at least six.

**Table 1:** Basic characteristics of the included studies

Authors	Year	Country	Male/Female	Age(years)	Definition of CAP	Surgical methods	Cough/Non-cough	Incidence rate (%)	Pathology	Operation method	TNM	Risk factor	NOS
Dong et al [13]	2022	China	50/48	-	Cough lasting no less than eight weeks with no obvious abnormalities present on chest X-ray	Lobectomy:69 Sublobectomy:29	31/67	31.6	-	VATS/OPEN	I-III	(2) (5) (6) (11) (12)	6
He et al [15]	2022	China	44/36	61.98 ± 3.48	(1) The patients developed cough within 30 days after surgery and lasted for more than 2 weeks; (2) No obvious cough history before surgery; (3) Cough caused by postoperative infection and other medical factors was excluded; (4) The chest CT scan revealed no significant abnormalities in the lung	Lobectomy:31 Others:49	20/60	25.0	NSCLC	VATS	I-II	(1) (5) (12) (13) (14)	8
Li et al [16]	2022	China	91/119	61.5(28–83)	Persistent dry cough for more than two weeks after surgery, without abnormalities in chest X-ray and blood count and other infection indicators, excluding cough caused by postnasal drip syndrome, bronchial asthma, oral ACEI drugs	Lobectomy:56; Wedge resection:154	72/138	34.3	-	VATS	0-III	(3) (5) (6) (10)	6
Ma et al [17]	2019	China	88/84	55.7 ± 11.88	-	Lobectomy:61 Segmentectomy:36 wedge resection:75	55/117	32.0	-	VATS	-	(4) (6) (11) (2)	6
Mu et al [4]	2017	China	319/331	59.7 ± 10.9	(1) No obvious cough history before surgery; (2) The patient developed cough within 30 days after surgery and lasted for more than two weeks; (3) Exclude tumor recurrence; (4) Exclude the cough caused by medical factors	Lobectomy:447 Sublobectomy:203	175/475	26.9	-	VATS	-	(2) (5) (6) (8) (12)	7
Yin et al [21]	2021	China	60/36	-	Postoperative cough lasting no less than eight weeks, persistent cough of unknown cause with dry cough as the main manifestation and no obvious abnormality on chest X-ray requiring medical intervention	Lobectomy:51 Sublobectomy:45	48/48	-	NSCLC	VATS	I-III	(5) (13) (15)	6
Zhang et al [22]	2022	China	71/49	-	Postoperative cough lasting no less than eight weeks, persistent cough of unknown cause with dry cough as the main manifestation and no obvious abnormalities on chest X-ray requiring medical intervention	Lobectomy:91 Segmentectomy:21 Wedge resection:8	67/53	55.8	NSCLC	VATS	I-IV	(1) (12) (13) (15)	7
Qian et al [18]	2021	China	123/135	-	(1) no obvious cough history before surgery; (2) postoperative cough occurred within 30 days after surgery and lasted no less than two weeks; (3) exclude tumor recurrence; (4) cough caused by postoperative infection and other medical factors was excluded	Lobectomy:45 Segmentectomy:29 Wedge resection:27	101/157	39.1	NSCLC	VATS	-	(3) (12)	7
Gao et al [14]	2022	China	71/57	63.2 ± 8.1	-	Segmentectomy	46/82	35.9	NSCLC	VATS	-	(1) (6) (12) (16) (17)	6
Wu et al [19]	2020	China	63/65	60.82 ± 9.89	Postoperative cough lasting no less than 8 weeks, persistent cough of unknown cause with dry cough as the main manifestation and no obvious abnormalities on chest X-ray requiring medical intervention	Lobectomy:88 Segmentectomy:18 Wedge resection:26	61/67	47.7	NSCLC	VATS/RATS	I-III	(5) (8) (13) (15)	6
Xin et al [20]	2021	China	279/291	57.7 ± 8.95	(1) No obvious cough history before surgery; (2) Exclude cough caused by internal diseases and oral drugs; (3) To exclude tumor recurrence in trachea or other sites; (4) Postoperative cough lasted for more than 2 weeks; (5) Postoperative imaging examination showed no abnormalities	Lobectomy	163/407	28.6	-	VATS	I-IV	(1) (2) (4) (6) (7) (8) (16)	7
Gu et al [8]	2022	China	79/62	-	Dry cough lasting no less than two weeks following pneumonectomy with no obvious abnormality present in a chest x-ray	Lobectomy:77 Sublobectomy:64	31/110	22.0	NSCLC	VATS	I	(1) (2) (4)	7
Lin et al [9]	2018	China	99/99	58.33 ± 9.69	-	Lobectomy:66 Sublobectomy:132	91/107	46.0	NSCLC	VATS	I-III	(1) (2) (3) (5) (7)	6
Lu et al [10]	2022	China	66/46	61.2 ± 9.8	(1) New-onset dry cough after lung resection, (2) Clear etiology with postnasal drip syndrome (PNDS) being excluded, (3) Cough lasting more than three weeks after surgery, (4) Normal blood tests and chest radiographs	Lobectomy	41/71	36.6	NSCLC	VATS/OPEN	-	(6)	7
Mu et al [3]	2022	China	445/456	58 (50–66)	(1) Cough occurring within two weeks after pulmonary resection; (2) Cough duration of no less than two weeks; and (3) Cough not caused by tumor recurrence or medical disease	Sublobectomy:435 Lobectomy or greater:466	190/711	21.1	-	VATS	-	(3) (6) (8) (18)	7
Pan et al [2]	2020	China	58/77	-	Dry cough that lasts no less than two weeks after pulmonary resection, except for nasal drip syndrome, bronchial asthma, or oral ACEI drugs, the chest X-ray revealed no apparent abnormalities	Lobectomy:55 Segmentectomy:12 Wedge resection:68	33/102	24.4	-	VATS	I-III	(1) (5) (7) (9)	7
Sawabata et al [11]	2005	Japan	38/32	64.7 ± 10.6	Nonproductive coughing that occurred more than two weeks after the operation with stable chest roentgenogram results, with no evidence of PNDS, asthma, or ACEI administration	Excision or segmentectomy:15 Lobectomy or greater:55	35/35	50.0	-	VATS/OPEN	-	(9) (18)	7
Wu et al [7]	2022	China	365/152	-	-	Lobectomy:293 Segmentectomy:143 wedge resection:81	207/310	40.0	-	VATS	-	(10) (14)	7
Xie et al [12]	2019	China	97/74	65(43–75)	A cough that lasts for more than eight weeks, primarily manifests as cough symptoms, has no abnormalities on X-ray, is not affected by conventional treatment, and has an unknown etiology	Lobectomy	68/103	39.8	NSCLC	VATS/OPEN	I-III	(2) (3) (6) (8) (17)	7

(1) anesthesia time; (2) right lobe operation; (3) gender; (4) upper lobe surgery; (5) lobectomy; (6) smoking history; (7) subcarinal lymph node dissection; (8) age; (9) postoperative acid reflux; (10) operation time; (11) closure of bronchial stump with stapler; (12) peritracheal lymph node resection; (13) tracheal intubation time; (14) drainage time; (15) preoperative cough; (16) BMI, body mass index; (17) COPD history; (18) mediastinal lymph node removal, *CAP* cough after pulmonary resection, *NSCLC* non-small cell lung cancer, *VATS* Video-assisted thoracoscopic surgery, *NOS* the Newcastle–Ottawa Quality Scale;—not reported, *CCS* case–control study, *RATS* robot-assisted thoracic surgery, *ACEI* angiotensin-converting enzyme inhibitors

The pooled results indicated that young age [OR = 0.66, 95% CI (0.46, 0.96), *p* = 0.03], female sex [OR = 1.69, 95% CI (1.07, 2.66), *p* = 0.02], preoperative cough [OR = 5.96, 95% CI (2.58, 13.73), *p* < 0.01], right lobe operation [OR = 2.14, 95% CI (1.44, 3.19), *p* < 0.01], lobectomy [OR = 3.70, 95% CI (1.73, 7.90), *p* < 0.01], subcarinal lymph node dissection [OR = 3.45, 95% CI (1.86, 6.39), *p* < 0.01], mediastinal lymph node removal [OR = 3.49, 95% CI (2.07, 5.89), *p* < 0.01], closure of bronchial stump with stapler [OR = 5.19, 95% CI (1.79, 15.07), *p* < 0.01], peritracheal lymph node resection [OR = 3.05, 95% CI (1.40,6.64), *p* < 0.01], postoperative acid reflux [OR = 11.07, 95% CI (4.38,28.02), *p* < 0.01] were independent risk factors for CAP. Smoking history, BMI, COPD history, upper lobe surgery, operation time, drainage time, anesthesia time, and tracheal intubation time were not associated with CAP. Publication bias test was not conducted because the number of studies included for each risk factor was less than ten (Table 2).

**Table 2:** Meta-analysis results of risk factors for cough after pulmonary resection

Risk factor	Operation method	Number of included studies	Heterogeneity		Model	Meta-analysis results	
			*P* value	*I*^*2*^ (%)		OR (95%CI)	*P* value
Age	VATS/OPEN	5 [3, 4, 12, 19, 20]	< 0.01	80	random	0.66 [0.46, 0.96]	**0.03**
	VATS	4 [3, 4, 19, 20]	< 0.01	84	random	0.66 [0.44, 1.00]	0.05
gender	VATS/OPEN	5 [3, 9, 12, 16, 18]	0.04	61	random	1.69 [1.07, 2.66]	**0.02**
	VATS	4 [3, 9, 16, 18]	0.07	57	random	1.95 [1.22, 3.11]	**< 0.01**
BMI	VATS	2 [14, 20]	< 0.01	89	random	0.78 [0.41, 1.47]	0.44
smoking history	VATS/OPEN	9 [3, 4, 10, 12–14, 16, 17, 20]	< 0.01	78	random	0.89 [0.56, 1.40]	0.60
	VATS	6 [3, 4, 14, 16, 17, 20]	< 0.01	68	random	0.62 [0.40, 0.96]	**0.03**
preoperative cough	VATS	2 [21, 22]	0.44	0	fixed	5.96 [2.58, 13.73]	**< 0.01**
COPD history	VATS/OPEN	2 [12, 14]	< 0.01	86	random	1.54 [0.19, 12.41]	0.68
upper lobe surgery	VATS	3 [8, 17, 20]	< 0.01	80	random	1.32 [0.45, 3.87]	0.61
right lobe operation	VATS/OPEN	7 [4, 8, 9, 12, 13, 17, 21]	0.09	46	random	2.14 [1.44, 3.19]	**< 0.01**
	VATS	5 [4, 8, 9, 17, 21]	0.05	59	random	1.91 [1.11, 3.29]	**0.02**
lobectomy	VATS/OPEN	7 [2, 4, 9, 13, 15, 16, 19]	< 0.01	81	random	3.70 [1.73, 7.90]	**< 0.01**
	VATS	6 [2, 4, 9, 15, 16, 19]	< 0.01	82	random	3.28 [1.48, 7.28]	**< 0.01**
subcarinal lymph node dissection	VATS/OPEN	3 [2, 9, 20]	0.77	0	fixed	3.45 [1.86, 6.39]	**< 0.01**
mediastinal lymph node removal	VATS/OPEN	2 [3, 11]	0.45	0	fixed	3.49 [2.07, 5.89]	**< 0.01**
closure of bronchial stump with stapler	VATS/OPEN	2 [13, 17]	0.93	0	random	5.19 [1.79, 15.07]	**< 0.01**
peritracheal lymph node resection	VATS/OPEN	6 [4, 13–15, 18, 22]	< 0.01	84	random	3.05 [1.40, 6.64]	**< 0.01**
	VATS	5 [4, 14, 15, 18, 22]	< 0.01	86	random	2.75 [1.24, 6.11]	**0.01**
operation time	VATS	2 [7, 16]	< 0.01	86	random	1.74 [0.50, 6.02]	0.38
drainage time	VATS	2 [7, 15]	< 0.01	92	random	1.60 [0.67, 3.84]	0.29
anesthesia time	VATS	7 [2, 8, 9, 14, 15, 20, 22]	< 0.01	91	random	1.02 [0.99, 1.04]	0.15
tracheal intubation time	VATS	3 [14, 20]	< 0.01	90	random	1.06 [0.97, 1.15]	0.21
postoperative acid reflux	VATS/OPEN	2 [2, 11]	0.72	0	fixed	11.07[4.38, 28.02]	**< 0.01**

*VATS* video-assisted thoracoscopic surgery, *BMI* body mass index, *COPD* chronic obstructive pulmonary disease, random, random-effects model; fixed, fixed-effects model

## Discussion

Persistent cough is a common postoperative complication following lung resection [1, 3]. With the development of ERAS, more attention is being paid to the quality of life of postoperative patients. Some studies have found that CAP can affect patients' postoperative quality of life, thus hindering their postoperative recovery [23]. Therefore, more and more researchers are actively studying CAP to accelerate the recovery of patients. Several risk factors have been shown to be associated with the occurrence of CAP [24]. To our knowledge, this is the first meta-analysis to investigate the risk factors for CAP. By including independent risk factors for CAP, our meta-analysis confirmed that young age, female sex, preoperative cough, right lobe surgery, lobectomy, subcarinal lymph node dissection, mediastinal lymph node removal, closure of bronchial stump with stapler, peritracheal lymph node resection, and postoperative acid reflux were independent risk factors for CAP. In the future, these identified risk factors can be used to construct predictive models to identify high-risk patients. For those high-risk patients, preoperative communication about CAP should be enhanced and intraoperative measures should be taken to prevent CAP, as well as more aggressive postoperative follow-up.

Our study showed that female sex was an independent risk factor for CAP. Previous studies have shown a preponderance of females with chronic cough, which may be related to the influence of female hormones [25, 26]. Back in 1989, researchers found that women who received angiotensin-converting enzyme inhibitors (ACEI) were more likely to cough than men [27]. Women have also been observed to be more sensitive than men to cough reflexes triggered by the inhalation of citric acid, tartaric acid and capsaicin [28–31]. C- fibers of the vagus nerve are the most important cough receptors and are mainly distributed in the larynx, trachea, carina and larger bronchi in the lung, which are sensitive to various chemical stimuli [32]. The ability of C fibers to sense chemical substances mainly depend on the expression of transient receptor potential (TRP) V1/A1 channels and other ion channels. Zhu et al. [33] discovered that the level of TRPV1 in patients with acute or chronic cough after lung cancer surgery was higher than that in patients without cough. Several studies have shown that estrogen can affect C fiber activation by affecting TRPV1 activation/sensitization [34]. Therefore, some researchers speculated that women's susceptibility to cough may be related to the influence of estrogen on TRPV1. In the future, drugs blocking the TRPV1 signaling pathway may be created to treat CAP. Interestingly, some studies have revealed that women were also at increased risk for chronic pain compared to men. Thus, some pain physiologists believed that women's greater susceptibility to chronic cough was part of an enhanced or overdeveloped visceral sensitivity that was the result of an evolutionary selection process [35]. Nonetheless, more relevant researches are needed to explore the mechanisms involved. A study indicated that the health-related quality of life (HRQOL) of women was more adversely affected than that of men, the longer a cough lasted [36]. Therefore, for female patients, more attention should be paid to cough and cough-related quality of life after lung cancer surgery. For female CAP patients, more aggressive treatments may be required.

Our study found that age was a risk factor for CAP, and younger people were more likely to develop CAP. This may be related to the relatively sluggish receptors of the cough reflex in the elderly [19]. However, age was not a risk factor for CAP when the surgical method was limited to thoracoscopic surgery, which is consistent with the conclusion of previous studies [3]. Due to the different age thresholds and few included studies, we were unable to determine which age group of patients were more likely to suffer CAP, future studies could further explore this. Our study found that longer anesthesia duration was not a risk factor for CAP, which was not consistent with previous studies [2, 8]. They thought the relationship between longer anesthesia duration and CAP may be due to the fact that the longer the time of tracheal intubation, the stronger the stimulation of the airway, resulting in a stronger inflammatory response of the tracheal tissue. However, in our study, cutoff values for anesthesia time varied across different studies, which may be a source of heterogeneity in our results. More research is warranted in the future to explore the association between duration of anesthesia and CAP. Previous studies have shown that patients with a history of smoking had less CAP after surgery, which was consistent with our research results. The reason may be that long-term smoking can reduce the sensitivity of airway cough receptors and weaken the sensitivity of cough reflex to the stimulation caused by surgery [37, 38]. Only two studies [21, 22] reported the relationship between preoperative cough and CAP, and the overall result indicated that preoperative cough was a risk factor for CAP. Airway hyperreactivity may be present in patients with cough before surgery. Therefore, appropriate drugs can be used preoperative to improve airway hyperreactivity in these patients.

Postoperative acid reflux was also an independent risk factor for CAP in current study, which was consistent with previous studies. This may be due to gastroesophageal reflux activate the vagus nerve from the esophagus to the lungs, as the vagus nerve innervates not only the bronchus but also the esophagus. However, the included studies had different definitions of postoperative acid reflux. Pan [2] et al. used the Reflux Diagnostic Questionnaire (RDQ) to assess the frequency and severity of reflux symptoms in postoperative patients. Postoperative acid reflux was diagnosed when the sum of the two scores was greater than 12. Sawabata [11] et al. diagnosed gastroesophageal reflux by asking patients if they had symptoms such as heartburn, nausea, chest pain and the characteristics of these symptoms. Thus, to further investigate the relationship between CAP and acid reflux, a 24-h esophageal pH monitor may be needed. Sawabata [11] et al. treated 20 CAP patients with acid reflux with proton pomp inhibiter and pro-kinetic agent and found significant improvement in cough in most patients. We therefore recommended that the RDQ could be used preoperatively to evaluate reflux. Proton pump inhibitors and prokinetic agents might be used to treat CAP patients with gastroesophageal reflux. The need for prophylactic use of proton pump inhibitors in patients with preoperative symptoms of reflux still requires further study.

The results of this meta-analysis showed that right lobe surgery, lobectomy, subcarinal lymph node dissection, mediastinal lymph node removal and peritracheal lymph node resection were risk factors for postoperative cough after pulmonary resection. Compared with segmentectomy or wedge resection, lobectomy contributes to a larger residual cavity in the thoracic cavity after surgery, which can lead to changes in the anatomical structure in the thoracic cavity, bronchial distortion, residual lung deformity, etc., thus increasing airway sensitivity and causing chronic cough [18]. Additionally, it was also associated with transecting a major bronchus while peforming lobectomy. According to this theory, patients undergoing pneumonectomy, which not only involves major bronchial transection but extensive dissection in the region of the carina, are more likely to develop CAP compared with patients undergoing lobectomy or sublobectomy, but this warrants further study due to the lack of relevant studies. The removal of lymph nodes may damage the vagus nerve, thus increasing the sensitivity of cough receptors and causing CAP. Of the studies we included, some evaluated the association between subcarinal lymph node dissection and CAP, some explored the association between paratracheal lymph node dissection and CAP, others investigated the relationship between mediastinal lymph node dissection and CAP. Therefore, we combined these three groups of data separately when performing the meta-analysis. Since mediastinal lymph nodes included several groups, further studies are needed to determine which group of lymph nodes dissection is more likely to cause CAP. Clarifying which group lymph node dissection is a risk factor for CAP may help us to understand the underlying mechanism. Our study suggested that compared with full-thickness interrupted suture, closure of bronchial stumps with stapler was a risk factor for CAP. Dong et al. speculated that closure of bronchial stump with stapler was not conducive to the discharge of airway secretions, and was prone to airway torsion or chronic inflammatory reaction of the airway stump, which may be the reason why closure of bronchial stump with stapler was an independent risk factor for CAP [13]. However, due to the inclusion of only two studies and the small percentage of bronchial stumps were closed with full-thickness interrupted suture, the result may be unstable and need to be confirmed by more high-quality studies.

Some researchers have conducted studies to explore how to prevent CAP from occurring. Dong et al. [13] found that preoperative lung training was a protective factor for CAP. Filling post-lymphadenectomy residual cavities with fatty tissue autografts has been shown to reduce the incidence of CAP while improving the quality of life of patients [39]. Gu et al. [8] discovered that intraoperative protection of pulmonary vagus nerve branches by sampling around the lymph nodes on the side of surgery reduced the incidence of CAP. Xie et al. [12] treated 41 CAP patients with acupuncture on the 8th week after surgery and found that the Leicester Cough Questionnaire in Mandarin Chinese (LCQ-MC) score was higher in the acupuncture treatment group compared to the no treatment group. Several studies have confirmed the efficacy of inhaled corticosteroid plus β2 agonist and the compound methoxyphenamine capsule for the treatment of CAP [6, 22]. Although some progress has been made, it is still worthwhile to further investigate who with CAP needs to be treated, and when and how to go about it. Our study identified several independent risk factors for CAP, which will provide some theoretical basis for the identification of patients at high risk for CAP, and the prevention and treatment of CAP in the future.

Wu et al. [19] observed no difference in the incidence of CAP between robot-assisted thoracic surgery (RATS) and video-assisted thoracic surgery (VATS). However, in the absence of relevant studies, we were unable to explore the differences in the incidence of CAP among thoracotomy, VATS, and RATS. Similarly, it is worthwhile to investigate whether there is any difference in the incidence of CAP between single-port and multi-ports VATS. Previously, we prospectively followed 88 post-thoracoscopic lung cancer patients and found that the incidence of CAP remained at 66% after 90 days after surgery, while the severity of the cough was gradually reduced [1]. Similar to our results, Lin et al. [23] found that the postoperative cough-related quality of life in lung cancer patients who underwent VATS returned to preoperative levels at approximately 3 months postoperative. However, there were still patients with cough symptoms at 6 months after surgery, who may require more attention. In this study, only one study included patients with cough that lasted longer than 90 days, they found that younger age (< 57 years), preoperative cough, lobectomy, and longer duration of endotracheal intubation (≥ 172 min) were risk factors for CAP [19]. In the future, more studies are needed to explore the characteristics of patients with prolonged postoperative cough.

There were several limitations in our study. First, all of the included studies were conducted in Asia, which may limit the applicability of the conclusions to other areas. Unfortunately, we were unable to find papers and data on CAP in regions other than Asia, and therefore, we were unable to provide specific information on CAP in other regions. Second, some proven risk factors, such as right upper lobectomy and difficult airway, could not be subjected to meta-analysis as they were reported in only one study. Hence, it is hoped that future research will give greater emphasis to the study of these risk factors. Third, some risk factors, such as age and anesthesia time, may lead to unstable conclusions due to different cutoff values adopted in different studies. We suggest that future studies could standardize these metrics. Fourth, CAP was inconsistently defined in different studies. Some studies consider CAP as postoperative cough lasting for at least two weeks, while others define it as lasting for a minimum of eight weeks. Moreover, certain studies did not provide a precise definition for CAP; rather, it was assessed by clinical physicians to determine whether patients experienced CAP. Therefore, there is a need to harmonize the definition of CAP in the future. Fifth, owing to the lack of relevant studies, we were unable to explain some of the underlying mechanisms. More in-depth studies are needed for the underlying mechanism of CAP. Given the number and quality of the included studies, more high-quality studies should be conducted to explore the risk factors for CAP, to better improve the quality of life of patients after lung resection.

In conclusion, young age, female sex, preoperative cough, right lobe surgery, lobectomy, subcarinal lymph node dissection, mediastinal lymph node removal, closure of bronchial stump with stapler, peritracheal lymph node resection, and postoperative acid reflux were independent risk factors for CAP. Patients with these risk factors may need more active intervention and postoperative follow-up to help them recover quickly and return to normal life.

## Data Availability

The used data sets analyzed during the study are available from the co-corresponding authors upon request.

## Abbreviations

OR: Odds ratio
CAP: Cough after pulmonary resection
ERAS: Enhanced recovery after surgery
NOS: The Newcastle–Ottawa Quality Scale
TRP: Transient receptor potential
HRQOL: Health-related quality of life
LCQ-MC: The Leicester Cough Questionnaire in Mandarin Chinese
RATS: Robot-assisted thoracic surgery
VATS: Video-assisted thoracic surgery
BMI: Body mass index
NSCLC: Non-small cell lung cancer
ACEI: Angiotensin-converting enzyme inhibitors
RDQ: Reflux Diagnostic Questionnaire
